# Early Acupuncture Following Thrombolysis in Acute Ischemic Stroke: A Meta‐Analysis of Randomized Controlled Trials on Efficacy and Safety

**DOI:** 10.1002/brb3.71840

**Published:** 2026-11-09

**Authors:** Geng Li, Yihao Zhou, Zhe He, Shuai Li, Qichen Bai, Shuochen Fan, Xu Chen, Mengqi Yue, Xiaolin Yang, Jing Shi

**Affiliations:** ^1^ Department of Acupuncture and Moxibustion Yunnan Provincial Hospital of Traditional Chinese Medicine Kunming Yunnan Province China; ^2^ The First Clinical Medical College Yunnan University of Chinese Medicine Kunming Yunnan Province China; ^3^ Department of Rehabilitation Medicine The Second Affiliated Hospital of Yunnan University of Chinese Medicine Kunming Yunnan Province China; ^4^ The Second Clinical Medical College Yunnan University of Chinese Medicine Kunming Yunnan Province China

**Keywords:** acute ischemic stroke, reperfusion injury, neuroprotection, NIHSS

## Abstract

**Introduction:**

Intravenous thrombolysis is standard for acute ischemic stroke (AIS), but many patients still have poor outcomes. Acupuncture may confer additional neuroprotection, yet its safety and efficacy when initiated early post‐thrombolysis remain unclear.

**Methods:**

A systematic search of databases and registries was conducted from inception to March 31, 2026, for randomized controlled trials comparing thrombolysis plus acupuncture with thrombolysis plus conventional post‐thrombolytic care in AIS patients within 14 days of onset. Meta‐analyses were performed using mean differences (MD) or risk ratios (RR) with 95% CIs. Heterogeneity was assessed with the *I* ^2^ statistic; publication bias was evaluated via Egger's test. Pre‐specified subgroup analyses were conducted.

**Results:**

Ten trials (1116 patients) were included. Adjunctive acupuncture reduced neurological deficit (NIHSS MD –2.90, 95% CI –3.62 to –2.17), improved activities of daily living (Barthel Index MD 12.07, 95% CI 8.10–16.03), and increased clinical response (RR 1.16, 95% CI 1.11–1.21; all *p* < 0.001). Adverse events did not differ significantly (RR 0.52, 95% CI 0.25–1.08). Only one study reported symptomatic intracranial hemorrhage (zero acupuncture vs. six control). Benefits were consistent across subgroups.

**Conclusion:**

In AIS patients receiving thrombolysis, acupuncture initiated early following thrombolysis improves neurological and functional outcomes. In available safety data, adverse events did not differ significantly between groups; however, systematic surveillance for symptomatic intracranial hemorrhage was lacking in most trials. These findings support further rigorous evaluation of early post‐thrombolysis acupuncture in adequately monitored randomized trials.

**Clinical Trial Registration:**

PROSPERO CRD420261358943

## Graphical abstract

This meta‐analysis included 10 randomized controlled trials involving 1,116 patients with acute ischemic stroke. Compared with thrombolysis plus standard care, adjunctive acupuncture was associated with reduced neurological deficits (NIHSS: MD = −2.90), improved daily living function (Barthel Index: MD = 12.07), and higher clinical response rates (RR = 1.16). No statistically significant difference in overall adverse events was observed (RR = 0.52). However, limited safety monitoring, particularly for symptomatic intracranial hemorrhage, warrants further investigation.

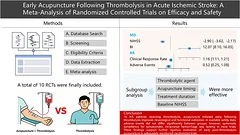

## 1 Introduction

In patients with acute ischemic stroke (AIS) who receive intravenous thrombolysis within the therapeutic window, successful recanalization often fails to yield favorable long‐term functional recovery. More than 50% of patients exhibit poor functional outcomes at 90 days despite imaging‐confirmed reperfusion (Shahid et al. 2022; X. Yang, Sun, et al. 2023). This disconnect is driven by secondary pathological cascades resulting from ischemia–reperfusion injury, notably increased blood–brain barrier permeability, heightened neuroinflammation, and mitochondrial dysfunction (Baron 2025; He et al. 2024; D. Q. Liu et al. 2024). Neuroprotective strategies targeting this post‐thrombolysis phase currently lack robust evidence (Zhao et al. 2024), establishing a critical clinical need for adjunctive interventions that can safely modulate these processes.

Preclinical studies have demonstrated that acupuncture can mitigate cerebral ischemia–reperfusion injury through multiple mechanisms, including modulation of autophagy (Tang et al. 2024), reduction of oxidative stress (Xia et al. 2025), limitation of neuroinflammation (W. Lin et al. 2026; Ren et al. 2024), modulation of apoptosis (Zhou et al. 2026), promotion of mitochondrial transfer (Guo et al. 2025), and decreased lactate accumulation (Xu et al. 2025). However, the clinical safety of applying acupuncture during the acute phase of thrombolytic therapy remains a principal concern limiting its broader adoption. Given the systemic effects of thrombolytic agents on coagulation, clinicians have expressed apprehension that needle manipulation might elevate the risk of subcutaneous hematoma or even exacerbate symptomatic intracranial hemorrhage (sICH) (McCulloch et al. 2015). Although preliminary clinical observations have not indicated a significant increase in bleeding events associated with acupuncture (Y. Li et al. 2022), this safety question demands rigorous systematic evaluation.

Previous systematic reviews and meta‐analyses of acupuncture for stroke have frequently employed broad inclusion criteria, aggregating data from patients across acute, subacute, and chronic phases, often without standardizing for concurrent thrombolysis (Luo et al. 2024; Z. Sun et al. 2025; S. Lin et al. 2022). Such methodological heterogeneity precludes direct extrapolation of their findings to the specific clinical scenario of acute‐phase adjunctive therapy with thrombolysis. Consequently, the net benefit of early acupuncture in this context remains unquantified. To address this gap, we conducted a meta‐analysis restricted to RCTs in which acupuncture was initiated in the early phase (within 14 days) following intravenous thrombolysis. While this time window encompasses both the hyperacute penumbra period and the early subacute phase, it reflects the range of current clinical practice and allows for the evaluation of acupuncture across the spectrum of early post‐thrombolytic care. Our primary objective was to quantify the effect of combined intervention on neurological deficit severity, with rates of sICH and serious adverse events as key safety endpoints. This report adheres to the PRISMA guidelines and aims to provide a quantitative evidence synthesis to inform early combined intervention strategies for AIS.

## 2 Methods

### 2.1 Study Registration and Reporting

This meta‐analysis was registered with PROSPERO (CRD420261358943). This systematic review and meta‐analysis was conducted in accordance with the Preferred Reporting Items for Systematic Reviews and Meta‐Analyses (PRISMA) 2020 statement. The completed PRISMA checklist is provided as Supporting Information.

### 2.2 Eligibility Criteria

**Population**: Adults aged 18–65 years with AIS confirmed by CT or MRI, treated within 14 days of symptom onset.

**Intervention**: Intravenous thrombolysis followed by any form of acupuncture initiated within 14 days of stroke onset.

**Control**: The same thrombolytic regimen followed by conventional post‐thrombolytic care (e.g., antiplatelet therapy, statins, and rehabilitation), without acupuncture.

**Outcomes**: The primary outcome was neurological deficit, measured by the National Institutes of Health Stroke Scale (NIHSS). Secondary outcomes included activities of daily living (Barthel Index [BI]), clinical response rate (defined as a binary outcome based on the degree of neurological improvement), and adverse events.

**Study Design**: Only randomized controlled trials (RCTs) were included.

### 2.3 Exclusion Criteria

Duplicate publications, reviews, animal studies, case reports, expert opinions, and studies without full text were excluded.

### 2.4 Search Strategy

We searched PubMed, Embase, Web of Science, Cochrane Library, Scopus, CNKI, Wanfang Data, VIP, Sinomed, and registries ChiCTR and CENTRAL from database inception to March 31, 2026. We used Medical Subject Headings and free‐text terms: “ischemic stroke,” “thrombolysis,” “acupuncture,” and “randomized controlled trial.” Reference lists of included studies were also screened. The detailed search strategy is provided in Supporting Information S1.

### 2.5 Study Selection and Data Extraction

Titles and abstracts were screened, full texts were assessed, and data were extracted independently by two reviewers using a standardized form. Discrepancies were resolved through discussion or consultation with a third reviewer. Missing data were requested from study authors where possible. Extracted information included study characteristics, participant demographics, intervention details, risk‐of‐bias assessment elements, and outcome data.

### 2.6 Risk of Bias Assessment

The methodological quality of included RCTs was assessed using the Cochrane Risk of Bias 2.0 (RoB 2.0) tool across five domains: randomization process, deviations from intended interventions, missing outcome data, outcome measurement, and selective reporting. Assessments were performed independently by two reviewers.

### 2.7 Statistical Analysis

All meta‐analyses were performed using R software (version 4.5.1). For continuous outcomes (NIHSS, BI, fibrinogen), mean differences (MD) were calculated. For one trial (Y. Y. Song et al. 2022) that reported NIHSS scores as medians and interquartile ranges, means and standard deviations were estimated using the formula proposed by Wan et al. (2014). Binary outcomes (clinical response rate and adverse events) were expressed as risk ratios (RR). All estimates are presented with 95% confidence intervals (CIs). Heterogeneity was assessed using Cochran's *Q* test (*p *< 0.10) and the *I* ^2^ statistic. Given the methodological diversity inherent in acupuncture trials, random‐effects models (with a restricted maximum likelihood estimator) were used for all analyses. Sensitivity analyses were performed using leave‐one‐out methods, and outliers were identified via Cook's distance and standardized residuals. A post hoc sensitivity analysis was performed excluding the trial for which means were mathematically estimated (Y. Y. Song et al. 2022), to verify that the pooled effect was not sensitive to data transformation. Trial sequential analysis (TSA) was performed for the primary outcome (NIHSS) using the O'Brien‐Fleming alpha‐spending function (two‐sided alpha = 0.05, beta = 0.20). The required information size was estimated assuming a clinically meaningful MD of −2.5 points on the NIHSS. The certainty of evidence was assessed using the GRADE framework for the primary and key secondary outcomes, and a summary of findings figure was constructed. Publication bias was assessed using funnel plots and Egger's regression test (*p *< 0.10 indicating asymmetry) when 10 or more studies were available. Pre‐specified subgroup analyses were conducted according to thrombolytic agent type (rt‐PA vs. urokinase), timing of acupuncture initiation (≤ 24 h vs. > 24 h vs. not specified), treatment duration (1, 2, or 4 weeks), and baseline NIHSS severity (5–15 vs. 16–20). All tests were two‐tailed, and *p* < 0.05 was considered statistically significant.

## 3 Results

### 3.1 Study Selection

The literature search yielded 624 records from databases and 16 from registries. After removing duplicates, 396 records underwent title/abstract screening, of which 385 were excluded. Eleven full‐text articles were assessed for eligibility, and one was excluded due to the unavailability of the full text. Ultimately, ten RCTs were included in the quantitative synthesis. The PRISMA flow diagram is shown in Figure 1.

**FIGURE 1:**
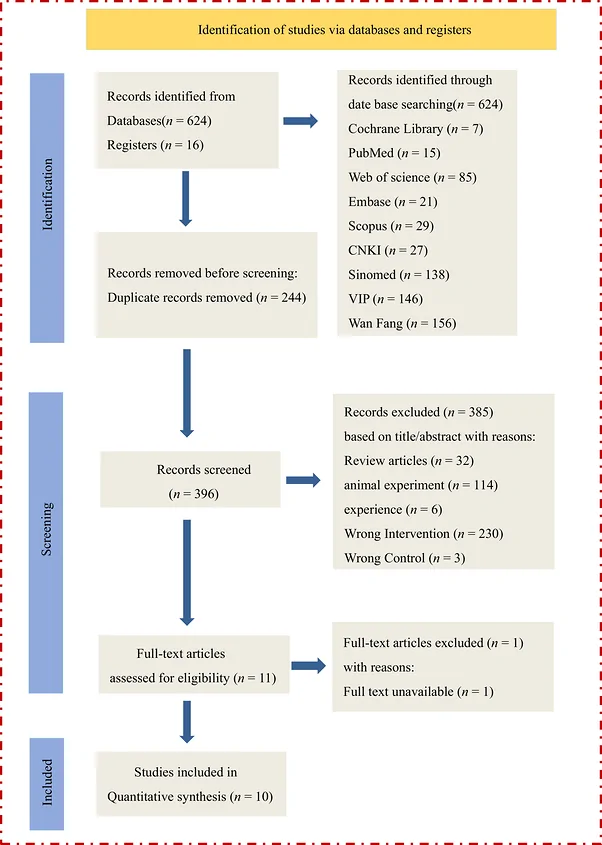
Study selection process according to the PRISMA 2020 guidelines. Of 640 records identified, 396 were screened after deduplication; 11 full‐text articles were assessed, and 10 RCTs were included in the quantitative synthesis.

### 3.2 Study Characteristics

Ten RCTs (2015–2023) with 1116 AIS patients (sample sizes: 40–360) were included, all from China. Patients in the intervention group received thrombolysis followed by acupuncture plus conventional care; those in the control group received thrombolysis followed by conventional care alone. Mean ages ranged from 51 to 64 years, with slightly more males. Six studies (Cao et al. 2020; Lu et al. 2023; L. J. Song et al. 2023; Y. Y. Song et al. 2022; J. Yang, Hua, et al. 2023; Q. Zhang and Tian 2020) detailed the time from symptom onset. NIHSS score was the primary outcome; secondary outcomes were BI, clinical response rate, adverse events, and fibrinogen levels. Seven studies (Cao et al. 2020; Lu et al. 2023; Y. Y. Song et al. 2022; Q. Zhang and Tian 2020; Chen et al. 2020; H. H. Liu et al. 2015; Q. Zhang et al. 2018) reported adverse events. Only Y. Y. Song et al. (2022) explicitly reported sICH (zero in the acupuncture group, six in the control group). Other adverse events were minor (gingival bleeding, ecchymosis, nausea, and dizziness), with no deaths or serious events. Study details are summarized in Table 1.

**TABLE 1:** Characteristics of included studies.

**Trial name**	**Simple size**	**Sex (male/female)**	**Age (years)**	**Disease course (mouth)**	**Therapeutic measures**	**Control measure**	**Outcomes MEASURES**	**Adverse reactions**
Liu et al., 2015	I: 50/50 C: 50/50	I: 36/14 C: 32/18	I: 54 C: 56.5	Not reported	UK + acupuncture	UK	NIHSS, BI, PT, APTT, PLT, Fib, response rate	I: 1 skin ecchymosis
Wei et al., 2023	I: 20/20 C: 20/20	I: 12/8 C: 11/9	I: 53.17 ± 6.15 C: 51.09 ± 7.22	Not reported	Rt‐PA + acupuncture	Rt‐PA	NIHSS, BI, FMA, mRS, response rate	Not reported
Song et al., 2022	I: 71/71 C: 70/71	I: 36/35 C: 37/33	I: 63 C: 64	I: 3.0 (1.5) h C: 3.0 (1.3) h	Rt‐PA + acupuncture	Rt‐PA	NIHSS, sICH Incidence, D‐dimer, Fib, PLT, response rate	I: 1 bleeding and 1 retinal hemorrhage C: 2 gingival bleeding, 1 perioral bleeding, and 6 cerebral hemorrhage
Cao et al., 2020	I: 54/54 C: 54/54	I: 32/22 C: 31/23	I: 56.3 ± 7.2 C: 57.2 ± 7.3	I: 29.1 ± 10.2 h C: 28.5 ± 9.71 h	Rt‐PA + acupuncture	Rt‐PA	NIHSS, Vp, Vm, DVp, DVm, PI, CVR, S‐100β, response rate	No adverse events.
Zhang et al., 2020	I: 50/50 C: 50/50	I: 29/21 C: 28/22	I: 56 ± 12 C: 55 ± 13	I: 2.68 ± 0.61 h C: 2.73 ± 0.64 h	Rt‐PA + acupuncture	Rt‐PA	NIHSS, MDA, LPO, CAT, SOD, CVR, PI, SS‐QOL, response rate	I: 2 gingival bleeding and 2 subcutaneous ecchymosis C: 2 nausea, 2 subcutaneous ecchymosis, and gingival bleeding
Zhang et al., 2018	I: 45/45 C: 45/45	I: 27/18 C: 26/19	I: 56.29 ± 7.20 C: 57.10 ± 7.29	Not reported	Rt‐PA + acupuncture	Rt‐PA	NIHSS, BI, response rate	No adverse events
Lu et al., 2023	I: 55/55 C: 55/55	I: 30/25 C: 32/23	I: 62.27 ± 15.76 C: 62.35 ± 15.84	I: 5.60 ± 0.61 d C: 5.58 ± 0.57 d	Rt‐PA + acupuncture	Rt‐PA	IL‐6, IL‐8, TNF‐α, NIHSS, BI, PV, Fib, HCT, response rate	I: 1 facial flushing, fever, and dizziness C: 3 gastrointestinal reactions and 1 headache
Chen et al., 2020	I: 54/54 C: 54/54	I: 31/23 C: 31/23	I: 56 ± 7 C: 57 ± 7	Not reported	Rt‐PA + acupuncture	Rt‐PA	NIHSS, CSS, BI, TEG, 5‐HT, E, DA, NE, response rate	C: 1 mild dizziness
Yang et al., 2023	I: 50/50 C: 50/50	I: 29/21 C: 26/24	I: 63.52 ± 1.47 C: 63.91 ± 1.55	I: 2.53 ± 0.61 h C: 2.70 ± 0.42 h	Rt‐PA + acupuncture	Rt‐PA	CRP, IL‐8, IL‐10, TNF‐α, RI, TTP, Qmean, CBV, SOD, MDA, Hcy, NIHSS, ADL, response rate	Not reported
Song et al., 2023	I: 180/180 C: 180/180	I: 92/88 C: 85/95	I: 56.68 ± 4.32 C: 57.11 ± 4.61	I: 3.58 ± 1.26 d C: 3.63 ± 1.36 d	Rt‐PA + acupuncture	Rt‐PA	NIHSS, BI, APTT, PT, Fib, response rate	Not reported

Abbreviations: 5‐HT, 5‐Hydroxytryptamine; APTT, activated partial thromboplastin time; BI, Barthel Index; C, control; CAT, catalase; CBV, cerebral blood volume; CRP, C‐reactive protein; CSS, Chinese Stroke Scale; CVR, cerebrovascular reserve; DA, dopamine; DVm, diastolic mean velocity; DVp, diastolic peak velocity; E, epinephrine; Fib, fibrinogen; FMA, Fugl‐Meyer Assessment; HCT, hematocrit; Hcy, homocysteineI, intervention; IL‐10, interleukin‐10; IL‐6, interleukin‐6; IL‐8, interleukin‐8; LPO, lipid peroxidation; MDA, malondialdehyde; mRS, modified Rankin Scale; NE, norepinephrine; NIHSS, National Institutes of Health Stroke Scale; PI, Pulsatility index; PLT, platelet count; PT, prothrombin time; PV, plasma viscosity; Qmean, mean blood flow; RI, Resistance Index; rt‐PA, recombinant tissue plasminogen activator; S‐100β, S‐100 calcium‐binding protein B; sICH, symptomatic intracranial hemorrhage; SOD, superoxide dismutase; SS‐QOL, Stroke‐Specific Quality of Life scale; TEG, thromboelastography; TNF‐α, tumor necrosis factor‐alpha; TTP, time to peak; UK, urokinase; Vm, mean velocity; Vp, peak velocity.

Regarding acupuncture timing, three studies (L. J. Song et al. 2023; H. H. Liu et al. 2015; Q. Zhang et al. 2018) initiated acupuncture within 24 h post‐thrombolysis, one study (Q. Zhang and Tian 2020) immediately after thrombolysis, and six studies (Cao et al. 2020; Lu et al. 2023; Y. Y. Song et al. 2022; J. Yang, Hua, et al. 2023; Chen et al. 2020; Wei et al. 2023) did not specify timing. Acupuncture sessions were mostly daily (except in one study [Y. Y. Song et al. 2022] with six sessions per week), with needle retention times of 20–30 min per session. Treatment duration ranged from 1 to 4 weeks, most commonly 2 weeks (Table 2).

**TABLE 2:** Description of the intervention.

**Trial Name**	**Timing of acupuncture intervention**	**Frequency of acupuncture**	**Acupuncture duration**	**Treatment duration**
Liu et al., 2015	24 h post‐thrombolysis	Once daily (7 times per week)	20 min	2 weeks
Wei et al., 2023	Not reported	Once daily (7 times per week)	30 min	4 weeks
Song et al., 2022	24 h post‐thrombolysis	Once daily (6 times per week)	30 min	1 weeks
Cao et al., 2020	Not reported	Once daily (7 times per week)	30 min	2 weeks
Zhang et al., 2020	post‐thrombolysis	Once daily (7 times per week)	30 min	2 weeks
Zhang et al., 2018	24 h post‐thrombolysis	Once daily (7 times per week)	30 min	2 weeks
Lu et al., 2023	Not reported	Once daily (7 times per week)	30 min	4 weeks
Chen et al., 2020	Not reported	Once daily (7 times per week)	30 min	4 weeks
Yang et al., 2023	Not reported	Once daily (7 times per week)	30 min	2 weeks
Song et al., 2023	Not reported	Once daily (7 times per week)	30 min	2 weeks

Reporting of concomitant pharmacological therapies was inconsistent across the 10 trials. Only two studies explicitly detailed specific agents and verified their balanced use between arms; one (Q. Zhang and Tian 2020) reported a protocol of aspirin, clopidogrel, and rosuvastatin, while the other (L. J. Song et al. 2023) confirmed that butylphthalide was administered to both groups. Two further studies partially described some agents without full clarity (Cao et al. 2020; Chen et al. 2020). The remaining six trials (Lu et al. 2023; Y. Y. Song et al. 2022; J. Yang, Hua, et al. 2023; H. H. Liu et al. 2015; Q. Zhang et al. 2018; Wei et al. 2023) either defined “conventional therapy” in vague terms (e.g., “antiplatelet,” “lipid‐lowering”) without specifying drugs, or did not report background pharmacotherapy at all. This widespread underreporting precludes a reliable assessment of the potential confounding introduced by co‐interventions (Table 3).

**TABLE 3:** Reporting quality of concomitant medications in included trials.

**Trial Name**	**Conventional therapy defined**	**Key concomitant medications reported**	**Balanced between arms**	**Treatment duration**
Liu et al., 2015	Partially, without specific agents	None	Unclear	Uncertain
Wei et al., 2023	Partially, without specific agents	None	Unclear	Uncertain
Song et al., 2022	Partially, without specific agents	None	Unclear	Uncertain
Cao et al., 2020	Partially, without specific agents	Aspirin: control group only	No	High
Zhang et al., 2020	Yes, explicitly listed	Aspirin, clopidogrel, rosuvastatin	Yes	Low
Zhang et al., 2018	Partially, without specific agents	Aspirin mentioned, unclear in both arms	Unclear	High
Lu et al., 2023	Not defined	None	Unclear	High
Chen et al., 2020	Partially, without specific agents	Aspirin: both arms	Yes	Moderate
Yang et al., 2023	Not defined	None	Unclear	High
Song et al., 2023	Yes, explicitly listed	Butylphthalide: both arms	Yes	Low

### 3.3 Risk of Bias

Using the RoB 2.0 tool, all studies were judged to have “some concerns” overall. Risks of missing outcome data and selective reporting were low across all trials. Eight studies had low risk of bias in the randomization process; two (L. J. Song et al. 2023; H. H. Liu et al. 2015) raised “some concerns” due to insufficient reporting of allocation concealment. All studies were rated as “some concerns” for deviations from intended interventions, as blinding of acupuncture practitioners is inherently infeasible. For outcome measurement, four studies (Y. Y. Song et al. 2022; Q. Zhang and Tian 2020; Chen et al. 2020; Wei et al. 2023) were at low risk, while the remaining six had “some concerns” due to unclear blinding of outcome assessors. No study was classified as high risk. As shown in Figure 2, all studies were judged to have “some concerns” overall. The proportion of trials rated as low risk or some concerns across each RoB 2.0 domain is presented in Figure 3.

**FIGURE 2:**
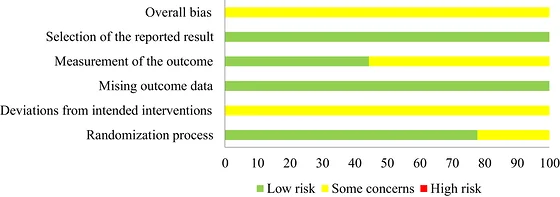
Risk of bias summary for the ten included RCTs assessed using the Cochrane Risk of Bias 2.0 tool across five domains: randomization process, deviations from intended interventions, missing outcome data, outcome measurement, and selective reporting. All studies were judged as “some concerns” overall.

**FIGURE 3:**
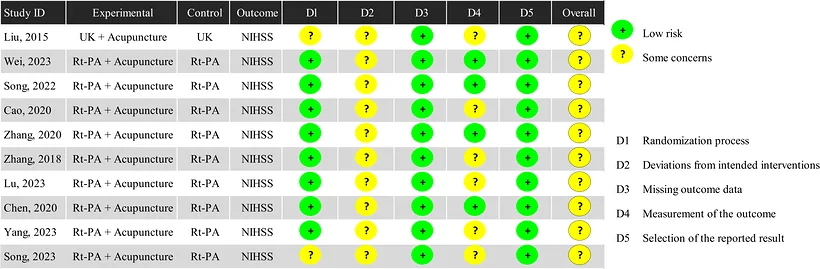
Risk of bias graph showing the proportion of trials rated as low risk, some concerns, or high risk across each RoB 2.0 domain.

### 3.4 Meta‐Analysis Findings

#### 3.4.1 NIHSS Score

All ten studies (*n* = 1116) reported NIHSS scores. The random‐effects meta‐analysis demonstrated that acupuncture plus thrombolysis significantly reduced NIHSS scores compared to thrombolysis alone (MD −2.90, 95% CI: −3.62 to −2.17; *p *< 0.001; GRADE certainty: low) (Figure 4A). Substantial heterogeneity was observed (*I^2^* = 94%). Sensitivity analysis identified J. Yang, Hua, et al. (2023) as an outlier (Cook's distance > 4/(k−2)). Upon exclusion of this study, the pooled MD was −2.51 (MD −2.51, 95% CI: −3.07 to −1.96; *p *< 0.001) with reduced heterogeneity (*I^2^ = *74%). Removal of any other single study did not materially alter the pooled estimate (Figure 4B). A further sensitivity analysis excluding the single trial for which means were estimated from medians and IQRs (Y. Y. Song et al. 2022) (MD −3.00, 95% CI: −3.94 to −2.07; *p *< 0.001), consistent with the primary analysis. The statistically and clinically significant benefit of acupuncture persisted, confirming that the overall finding was not driven by data transformation artifacts. TSA confirmed that the cumulative *Z*‐score (−26.76) crossed the O'Brien‐Fleming sequential monitoring boundary (1.96 at the final analysis), indicating sufficient sample size to support the observed benefit with a low risk of false‐positive error (Supporting Information).

**FIGURE 4:**
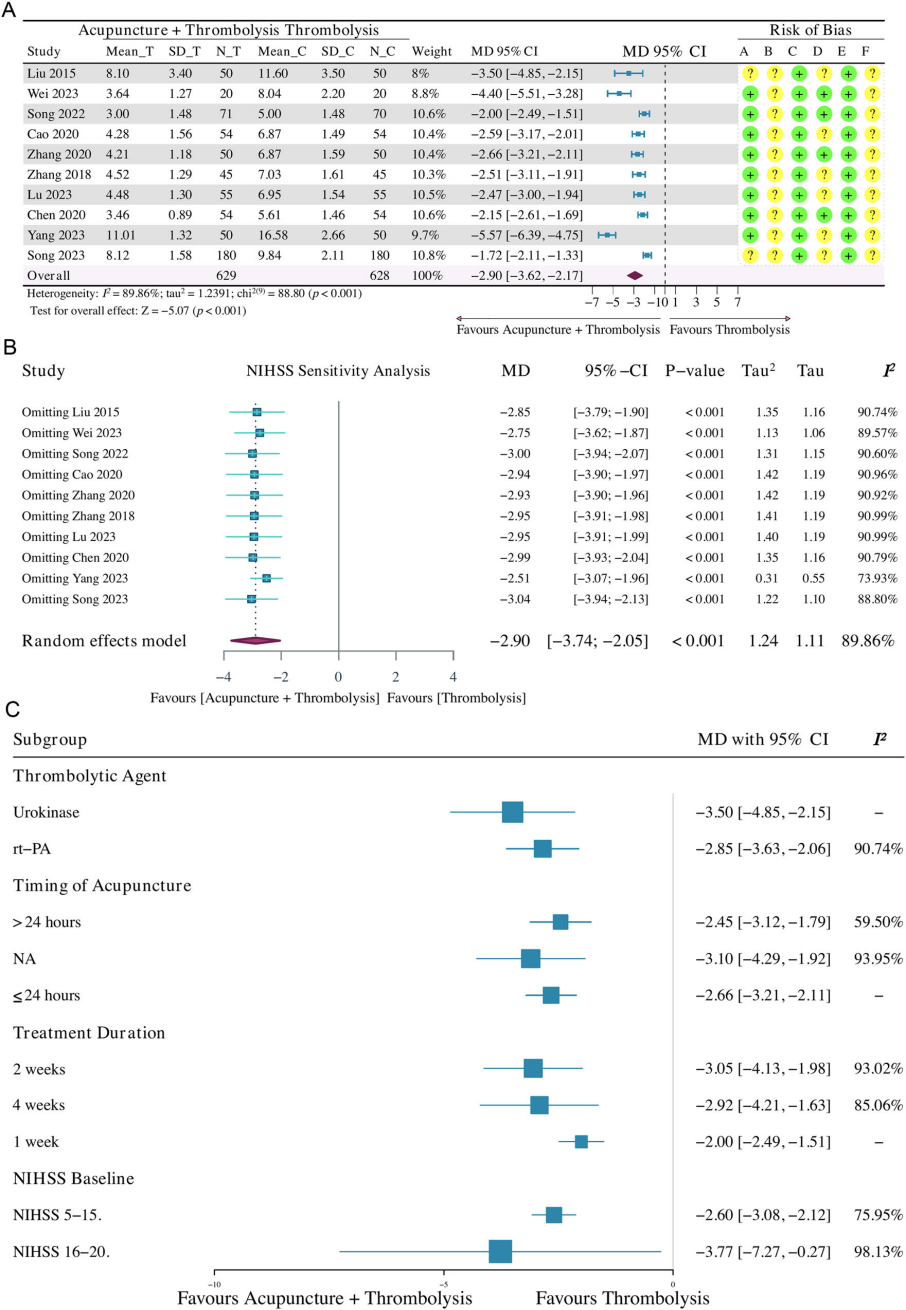
Meta‐analysis of NIHSS scores comparing acupuncture plus thrombolysis with thrombolysis plus conventional care. (A) Forest plot of random‐effects meta‐analysis (MD re plus thrombolysis with thro*p* < 0.001, *I* ^2^.001, p*n* = 1116). (B) Leave‐one‐out sensitivity analysis showing the influence of individual study exclusion on the pooled MD. (C) Subgroup analyses stratified by thrombolytic agent, timing of acupuncture initiation, treatment duration, and baseline stroke severity (all *p* < 0.05 for subgroup effects).

Subgroup analyses stratified by thrombolytic agent (rt‐PA: MD −2.85, 95% CI: −3.33 to −2.06; urokinase: MD −3.50, 95% CI: −4.85 to −2.15), acupuncture timing (≤ 24h: MD −2.66, 95% CI: −3.21 to −2.11; > 24h: MD −2.45, 95% CI: −3.12 to −1.79; no information (NA): MD −3.10, 95% CI: −4.29 to −1.92), treatment duration (1 week: MD −2.00, 95% CI: −2.49 to −1.51; 2 weeks: MD −3.05, 95% CI: −4.13 to −1.98; > 2 weeks: MD −2.92, 95% CI: −4.21 to −1.63), and baseline NIHSS severity (5–15: MD −2.60, 95% CI: −3.08 to −2.12; 16–20: MD −3.77, 95% CI: −7.27 to −0.27) (Figure 4C). The point estimate favored acupuncture in both strata; however, the severe subgroup contained only two trials (Y. Y. Song et al. 2022; J. Yang, Hua, et al. 2023), yielding a wide CI that precludes definitive inference for patients with severe baseline deficits.

#### 3.4.2 Barthel Index

Seven studies (Lu et al. 2023; L. J. Song et al. 2023; J. Yang, Hua, et al. 2023; Chen et al. 2020; H. H. Liu et al. 2015; Q. Zhang et al. 2018; Wei et al. 2023) (*n* = 908) reported BI scores. In a random‐effects meta‐analysis, adjunctive acupuncture demonstrated a significant improvement in BI (MD 12.07, 95% CI: 8.10–16.03; *p* < 0.001; GRADE certainty: very low) (Figure 5A). However, high heterogeneity was present. Notably, leave‐one‐out analysis did not identify any influential outliers, and the pooled estimates remained stable (range 10.99–13.02) and statistically significant (Figure 5B).

**FIGURE 5:**
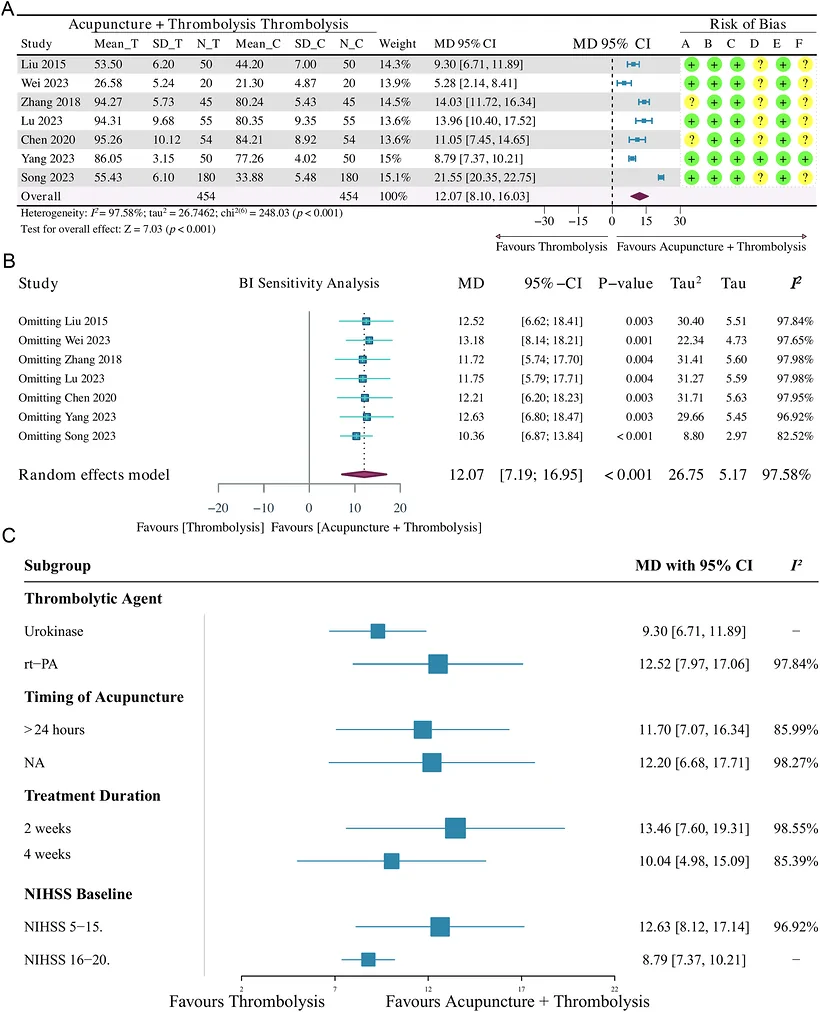
Meta‐analysis of Barthel Index scores comparing acupuncture plus thrombolysis with thrombolysis plus conventional care. (A) Forest plot of random‐effects meta‐analysis (MD 12.07, 95% CI 8.10–16.03, *p* < 0.001, *I* ^2^ = 97.58%, *n* = 908). (B) Leave‐one‐out sensitivity analysis showing the influence of individual study exclusion on the pooled MD. (C) Subgroup analyses stratified by thrombolytic agent, timing of acupuncture initiation, treatment duration, and baseline stroke severity (all *p* < 0.05 for subgroup effects).

In addition, subgroup analyses demonstrated consistent benefits across thrombolytic agent type (rt‐PA: MD 12.52, 95% CI: 7.97–17.06; urokinase: MD 9.30, 95% CI: 6.71–11.89), acupuncture timing (> 24h: MD 11.70, 95% CI: 7.07–16.34; NA: MD 12.20, 95% CI: 6.68–17.71), treatment duration (2 weeks: MD 13.46, 95% CI: 7.60–19.31; 4 weeks: MD 10.04, 95% CI: 4.98–15.09), and baseline NIHSS (5–15: MD 12.63, 95% CI: 8.12–17.14; 16–20: MD 8.79, 95% CI: 7.37–10.21) (Figure 5C).

#### 3.4.3 Clinical Response Rate

All ten studies reported clinical response rates. A random‐effects model revealed a significantly higher response rate in the acupuncture group (RR 1.16, 95% CI: 1.11 to 1.21; *p* < 0.001; GRADE certainty: moderate), with no evidence of heterogeneity (*I^2^* = 0%, *p* = 0.56) (Figure 6).

**FIGURE 6:**
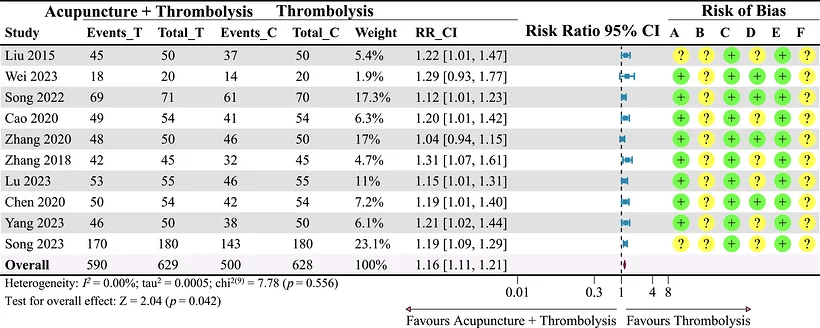
Forest plot of random‐effects meta‐analysis comparing clinical response rates between groups (RR 1.16, 95% CI 1.11 to 1.21, *p* < 0.001, *I* ^2^ = 0%, *n* = 1116).

#### 3.4.4 Adverse Events

Five studies (Lu et al. 2023; Y. Y. Song et al. 2022; Q. Zhang and Tian 2020; Chen et al. 2020; H. H. Liu et al. 2015) (*n* = 559) reported adverse events. A random‐effects meta‐analysis showed no statistically significant difference in adverse event incidence between groups (RR 0.52, 95% CI: 0.25–1.08; *p* = 0.08; GRADE certainty: low), with no heterogeneity (*I^2^* = 0%, *p = *0.59) (Figure 7). One additional study (Cao et al. 2020) reported no events in either arm.

**FIGURE 7:**
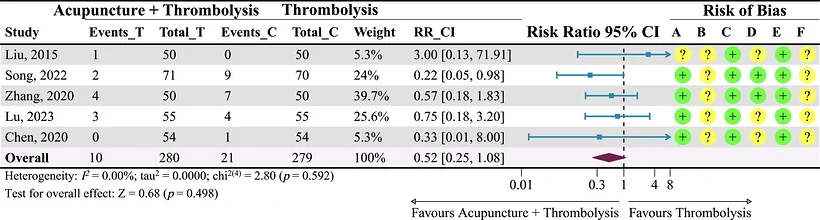
Forest plot of random‐effects meta‐analysis comparing adverse event rates between groups (RR 0.52, 95% CI 0.25 to 1.08, *p* = 0.08, *I* ^2^ = 0%, *n* = 559).

### 3.5 Publication Bias

Visual inspection of the funnel plot did not reveal marked asymmetry (Figure 8). Egger's test suggested possible funnel plot asymmetry (t = 1.90, P = 0.0936), although this finding should be interpreted cautiously given the limited number of studies. suggesting no substantial publication bias. These pooled estimates reflect an absence of detected safety signals under incomplete monitoring rather than verified protection against bleeding risks.

**FIGURE 8:**
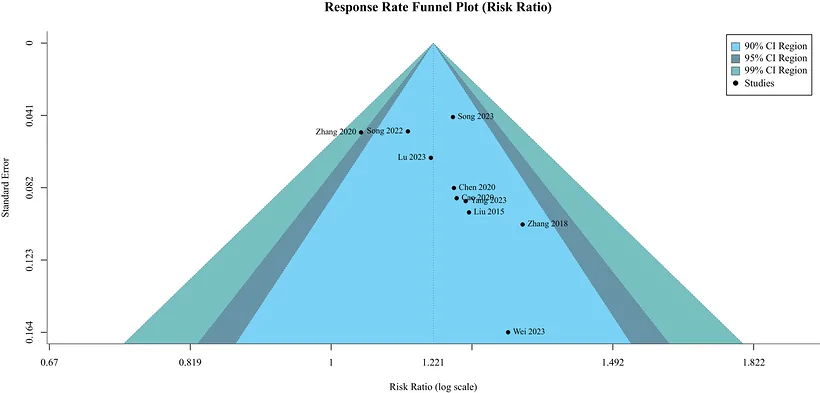
Funnel plot for assessment of publication bias across studies reporting NIHSS scores. EggerR 0.52, 9*t* = 1.90, *p* = 0.0936.

### 3.6 Certainty of Evidence

By the GRADE framework, no outcome achieved high certainty (Figure 9). Efficacy evidence was rated as low for NIHSS and very low for the BI, both downgraded for risk of bias and inconsistency; clinical response rate was rated as moderate, downgraded for risk of bias from unblinded assessment of a subjective endpoint. Safety evidence was rated as low for adverse events, downgraded for risk of bias from passive reporting and imprecision. These ratings underscore that the safety profile of early post‐thrombolysis acupuncture remains inadequately characterized, and that future trials should address the specific downgrading factors identified here—particularly the lack of blinding, the incomplete safety surveillance, and the absence of long‐term functional outcomes.

**FIGURE 9:**
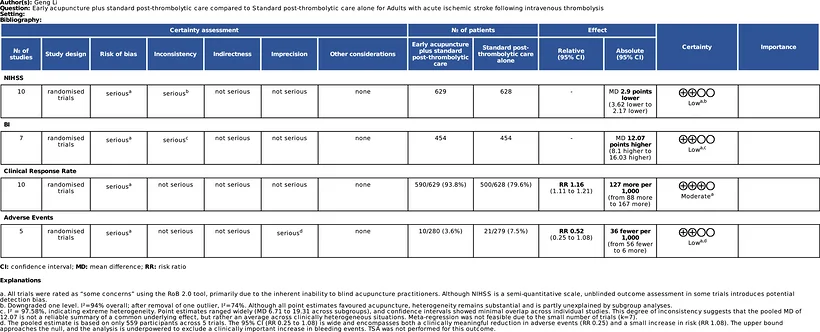
GRADE evidence profile summarizing the certainty of evidence for primary and key secondary outcomes. Certainty was rated as low for NIHSS (downgraded for risk of bias and inconsistency), very low for Barthel Index (risk of bias and inconsistency), moderate for clinical response rate (risk of bias), and low for adverse events (risk of bias and imprecision).

## 4 Discussion

### 4.1 Principal Findings

This meta‐analysis provides the first quantitative synthesis of the safety and efficacy of initiating acupuncture in the early days following thrombolysis for AIS. Owing to previous evidence gaps, clinicians have often deferred acupuncture to subacute or recovery phases (H. Jiang et al. 2024). Our analysis of 10 RCTs comprising 1116 patients demonstrates that adjunctive acupuncture significantly reduces NIHSS scores (MD −2.90, 95% CI: −3.62 to −2.17), improves BI scores (MD 12.07, 95% CI: 8.10–16.03), and enhances clinical response rates (RR 1.16, 95% CI: 1.11–1.21). Available safety data, derived from five of the ten trials, showed no statistically significant increase in overall adverse events (RR 0.52, 95% CI: 0.25–1.08). However, these data must be interpreted cautiously: only one study (Y. Y. Song et al. 2022) implemented systematic sICH surveillance, and the remaining trials provided only passive reporting of adverse events. The absence of protocol‐defined safety monitoring in most studies precludes definitive conclusions regarding bleeding risk. These findings indicate that early acupuncture initiated post‐thrombolysis provides an independent, clinically meaningful benefit for neurological and functional recovery when added to thrombolysis. Subgroup analyses confirmed directionally consistent benefits across thrombolytic agents, timing of acupuncture initiation, and treatment duration. The benefit in the severe stroke subgroup (NIHSS 16–20) was nominally significant but derived from only two trials (Y. Y. Song et al. 2022; J. Yang, Hua, et al. 2023), yielding a wide CI that precludes definitive conclusions for this population. The observation that benefit was preserved regardless of whether acupuncture began within or beyond 24 h suggests that both hyperacute neuroprotection and later‐phase repair mechanisms may contribute, although the present aggregate data cannot distinguish these temporally distinct effects.

Regarding intracranial safety, only one study (Y. Y. Song et al. 2022) systematically reported sICH, documenting zero events in the acupuncture group versus six in the control group. While this observation, together with preclinical evidence suggesting that acupuncture may mitigate hemorrhagic transformation via multiple mechanisms (Z. Huang et al. 2025; T. Jiang et al. 2025; Z.‐H. Zhang et al. 2025) and a supportive real‐world cohort study (Liang et al. 2023), surveillance in the other nine trials precludes a pooled risk estimate.

This marked imbalance warrants cautious interpretation. Acupuncture has no known antifibrinolytic or direct hemostatic effect capable of eliminating thrombolysis‐related intracranial bleeding, making a true protective effect of this magnitude biologically implausible. Potential contributors include unrecognized baseline prognostic imbalances despite randomization, differential ascertainment of neurological deterioration in an unblinded trial, and the statistical compatibility of zero events with a low underlying event rate in a modest sample. This observation should therefore be regarded as a hypothesis‐generating signal rather than confirmatory evidence of protection. More broadly, the pooled safety estimates in this review reflect an absence of detected signals under incomplete monitoring, not verified protection against bleeding risks. Definitive assessment of intracranial safety requires future trials with protocol‐mandated neuroimaging and blinded endpoint adjudication.

### 4.2 Heterogeneity and Subgroup Analyses

Substantial heterogeneity was observed in the NIHSS analysis (*I^2^* = 94%), driven largely by two studies enrolling patients with more severe baseline deficits (NIHSS > 15) (Y. Y. Song et al. 2022; J. Yang, Hua, et al. 2023), whose pooled effect (MD –3.77) exceeded that of the remaining eight studies (MD –2.60). This severity‐dependent pattern is consistent with observations from thrombolysis and thrombectomy trials, in which patients with greater baseline impairment derive a larger absolute benefit (Escudero‐Martínez et al. 2023). In the urokinase subgroup, the effect size (MD –3.50) was numerically larger than that for rt‐PA (MD –2.85), possibly reflecting the selection of patients with slower stroke progression and more robust collateral compensation, given the extended time window for urokinase administration (F. Sun et al. 2020). Benefit was evident across both early (≤ 24 h) and unspecified timing subgroups, suggesting efficacy even with delayed initiation. In the treatment duration subgroup, the largest effect was observed for the 2‐week regimen (MD –3.05), with smaller estimates for both shorter and longer courses. This pattern may reflect the temporal dynamics of endogenous neuroplasticity following stroke—synaptic plasticity in the human brain has been shown to peak approximately 2 weeks after ischemic stroke (Hordacre et al. 2021), and a 2‐week acupuncture regimen may coincide with this period of heightened plasticity. These subgroup findings are derived from study‐level data with limited trials in certain strata and are susceptible to ecological bias; they should therefore be regarded as hypothesis‐generating. Meta‐regression was not performed, as the small number of trials (*k* = 10) provided insufficient power for reliable moderator analysis. Consequently, confounding from unbalanced baseline stroke severity cannot be excluded as a contributor to the observed heterogeneity. The residual heterogeneity (*I* ^2^ = 74% after exclusion of J. Yang, Hua, et al. (2023)) may further reflect methodological diversity—including variations in acupuncture type, acupoint selection, and needling technique—that study‐level covariates cannot capture. The residual heterogeneity may also reflect clinically meaningful differences in acupuncture protocols. For example, the Xingnao Kaiqiao protocol used in most trials and the Tongdu Tiaoshen approach used in Wei et al. (2023) differ substantially in acupoint selection, needling technique, and treatment frequency, yet they were pooled under a single category. Formal comparison by acupuncture style was precluded by the small number of trials per category and the risk of ecological bias. The pooled effect estimate should therefore be interpreted as an average response across heterogeneous clinical settings rather than a single, universally applicable treatment effect.

### 4.3 Comparison With Previous Work

To our knowledge, this is the first meta‐analysis specifically evaluating the efficacy and safety of acupuncture initiated during the acute post‐thrombolysis period in AIS. A previous meta‐analysis of 93,057 patients with minor stroke demonstrated that intravenous thrombolysis alone improves discharge NIHSS (OR 2.19, 95% CI 1.56–3.08) (Moawad et al. 2025). In the present analysis, the addition of acupuncture was associated with a 2.90‐point reduction in NIHSS (95% CI –3.62 to –2.17), suggesting incremental neurological benefit beyond thrombolysis alone. Compared with prior meta‐analyses of acupuncture for stroke (Luo et al. 2024; Z. Sun et al. 2025; S. Lin et al. 2022; S. Huang et al. 2024; S. Li et al. 2026; Zhu et al. 2024), this study differs in three respects. First, previous reviews aggregated patients across all stroke stages, excluding those who received thrombolysis, limiting applicability to the high‐risk early post‐thrombolysis window. Second, whereas prior pooled NIHSS estimates range from –1.70 to –4.12 (S. Huang et al. 2024; S. Li et al. 2026; Zhu et al. 2024), our estimate of –2.90 reflects a homogeneous population treated with thrombolysis. Third, sICH—the most serious complication of thrombolysis—has not been systematically evaluated in prior syntheses; we pre‐specified this outcome and observed no signal of increased risk (RR 0.52). Collectively, these findings indicate that acupuncture provides an independent, quantifiable clinical benefit when added to standard thrombolytic therapy, addressing key gaps in population specificity and safety assessment.

### 4.4 Strengths and Limitations

Strengths of this meta‐analysis include its methodological rigor: adherence to PRISMA guidelines, prospective registration, comprehensive search strategy across Chinese and English databases and trial registries, use of the RoB 2.0 tool, and pre‐specified subgroup and sensitivity analyses. The NIHSS, a well‐validated and widely used measure of neurological deficit strongly correlated with infarct volume and functional outcomes, served as the primary endpoint, ensuring comparability across studies (F. Sun et al. 2020; Hordacre et al. 2021).

Several limitations warrant consideration. First, blinding of acupuncture practitioners was not possible in any of the included trials, introducing potential performance bias. However, the semi‐quantitative nature of the NIHSS and blinding of outcome assessors in several studies mitigate some concerns regarding detection bias. Second, the reporting of conventional post‐thrombolytic care—including specific antiplatelet agents, statins, neuroprotective drugs, and structured rehabilitation protocols—was highly inconsistent across trials, as detailed in Section 2.2. The control group in all included trials was not “thrombolysis alone” but rather thrombolysis followed by these routine therapies; therefore, the observed effect of acupuncture represents an incremental benefit above this variable background, and the magnitude of this increment may be influenced by imbalances in co‐interventions. Third, the safety analysis is constrained by substantial missing data. Only five of 10 trials contributed to the adverse event meta‐analysis, and systematic sICH surveillance with protocol‐defined neuroimaging was implemented in only one study. Consequently, the observed RR for adverse events (RR 0.52) and the absence of reported sICH in the acupuncture arm cannot be interpreted as evidence of safety, but rather as an absence of detected safety signals under incomplete monitoring. Fourth, the evidence base lacks long‐term functional outcome data. The 90‐day modified Rankin Scale (mRS) is the accepted gold standard in stroke trials. Of the 10 included studies, only one reported 90‐day mRS outcomes (Wei et al. 2023). Our primary endpoint, NIHSS at discharge or short‐term follow‐up, captures neurological deficit rather than functional recovery, and acute improvements on this scale do not necessarily translate into sustained functional independence over time. The absence of 90‐day mRS data in the remaining nine trials substantially limits the clinical applicability of our findings. In addition, six of the 10 included trials did not specify the interval between thrombolysis and acupuncture initiation. This lack of temporal information precludes meaningful stratification of ultra‐early versus delayed intervention, and the pooled estimate therefore combines data from pathophysiologically distinct therapeutic windows. We acknowledge that this temporal heterogeneity substantially limits the clinical applicability of the pooled estimates. Consequently, no clinical recommendation regarding the optimal timing of acupuncture initiation can be derived from the present findings. Finally, all 10 included trials were conducted exclusively in China. Although this reflects the current concentration of clinical research on post‐thrombolysis acupuncture in China, the findings may not be directly generalizable to international stroke centers with differing post‐thrombolytic care pathways and patient populations. Multinational trials are needed to validate these results across diverse healthcare settings. In summary, these methodological gaps—open‐label designs, passive safety reporting, and incomplete documentation of background therapies—introduce detection and performance biases that statistical methods cannot resolve. Future trials should therefore adopt rigorous blinding, protocol‐defined safety surveillance, and standardized concomitant care.

### 4.5 Clinical Implications and Future Directions

The current evidence base is insufficient to support firm clinical recommendations regarding the optimal timing, duration, or protocol of post‐thrombolysis acupuncture. Although our subgroup analysis found that benefit was evident when acupuncture was initiated within 24 h of thrombolysis, this finding is derived from only a subset of trials and should be regarded as hypothesis‐generating. In clinical practice, acupuncture is generally initiated only after hemodynamic stabilization, and the safety data in our review remain too sparse to endorse ultra‐early intervention. The provisional parameters observed across the included studies may inform the design of future trials, but they do not yet constitute evidence‐based recommendations. Large‐scale, multicenter, prospectively registered RCTs with rigorous safety monitoring and standardized reporting of concomitant therapies are urgently needed before clinical guidance can be formulated. Future studies should prioritize the use of core outcome sets, with mandatory reporting of 90‐day mRS scores, protocol‐defined sICH, and serious adverse events. Acupuncture protocols—including point selection, needle retention time, frequency, and total duration—should be standardized and reported in sufficient detail to enable individual participant data meta‐analyses and heterogeneity characterization in future syntheses. In addition, individual participant data meta‐analyses are encouraged to identify patient subgroups most likely to benefit (e.g., those stratified by age, baseline stroke severity, and thrombolytic agent).

## 5 Conclusion

In patients with AIS, acupuncture initiated early after intravenous thrombolysis clearly reduces neurological deficit, improves functional independence, and boosts clinical response rate compared with standard post‐thrombolytic care alone. In the limited available safety data, adverse events did not differ significantly between groups, although systematic surveillance for sICH was lacking in most trials. Until more definitive prospective trials with rigorous safety monitoring are available, early acupuncture should be evaluated further before it can be recommended for routine clinical use.

## Author Contributions

**Geng Li**: conceptualization, methodology, data curation, formal analysis, writing – original draft. **Yihao Zhou**: investigation, writing – original draft, validation. **Zhe He**: data curation. **Shuai Li**: data curation. **Qichen Bai**: data curation. **Shuochen Fan**: visualization. **Xu Chen**: software, resources. **Mengqi Yue**: project administration. **Xiaolin Yang**: supervision. **Jing Shi**: funding acquisition, writing – review and editing. All authors read and approved the final manuscript.

## Funding

This work was supported by the Basic Research Key Project of Yunnan Provincial Department of Science and Technology (Grant No. 202101AZ070001‐165); the “Xingdian Talents Support Program” Famous Doctor Special Project of Yunnan Province (Yun Cai She [2026] No. 1, 2025 Famous Doctor Talent Special Fund); and the Key Project of Joint Fund of Yunnan University of Chinese Medicine and Affiliated Hospitals (Grant No. XYLH2024021).

## Ethics Statement

This article does not contain any studies with human participants or animals performed by any of the authors.

## Consent

Not applicable because this study is a meta‐analysis of previously published data.

## Conflicts of Interest

The authors declare no conflicts of interest.

## Supporting information




**Supplementary Material**: brb371840‐sup‐0001‐SuppMat.pdf

**Supplementary Material**: brb371840‐sup‐0002‐SuppMat.docx

**Supplementary Material**: brb371840‐sup‐0003‐SuppMat.docx

**Supplementary Material**: brb371840‐sup‐0004‐SuppMat.docx

## Data Availability

All data generated or analyzed during this study are included in this published article and its Supporting Information files.
