# Supplementary material for: Early Acupuncture Following Thrombolysis in Acute Ischemic Stroke: A Meta‐Analysis of Randomized Controlled Trials on Efficacy and Safety
Source: Brain Behav. 2026 Nov 9;16(11):e71840. doi: 10.1002/brb3.71840 (PMC13746989; doi:10.1002/brb3.71840)
Supplement: Supplementary file 1 [file BRB3-16-e71840-s002.pdf]

Search strategy

Pubmed

("stroke"[MeSH Terms] OR "brain ischemia"[MeSH Terms] OR "ischemic stroke"[Title/Abstract] OR "cerebral infarction"[Title/Abstract] OR "cerebral ischemia"[Title/Abstract] OR "brain infarction"[Title/Abstract] OR "cerebrovascular accident"[Title/Abstract])

AND

("tissue plasminogen activator"[MeSH Terms] OR "thrombolytic therapy"[MeSH Terms] OR "alteplase"[Title/Abstract] OR "rt-PA"[Title/Abstract] OR "tenecteplase"[Title/Abstract] OR "reteplase"[Title/Abstract] OR "urokinase"[Title/Abstract] OR "streptokinase"[Title/Abstract] OR "thrombolysis"[Title/Abstract])

AND

("acupuncture"[MeSH Terms] OR "acupuncture therapy"[MeSH Terms] OR "electroacupuncture"[MeSH Terms] OR "acupuncture"[Title/Abstract] OR "electroacupuncture"[Title/Abstract] OR "electro-acupuncture"[Title/Abstract] OR "needling"[Title/Abstract] OR "scalp acupuncture"[Title/Abstract] OR "body acupuncture"[Title/Abstract] OR "abdominal acupuncture"[Title/Abstract])

AND

("randomized controlled trial"[Publication Type] OR "controlled clinical trial"[Publication Type] OR "randomized"[Title/Abstract] OR "randomised"[Title/Abstract] OR "placebo"[Title/Abstract] OR "randomly"[Title/Abstract] OR "trial"[Title/Abstract])

Scopus

(TITLE-ABS-KEY (stroke OR "brain ischemia" OR "ischemic stroke" OR "cerebral infarction" OR "cerebral ischemia" OR "brain infarction" OR "cerebrovascular accident"))

AND

(TITLE-ABS-KEY ("tissue plasminogen activator" OR alteplase OR "rt-PA" OR tenecteplase OR reteplase OR urokinase OR streptokinase OR thrombolysis OR "thrombolytic therapy"))

AND

(TITLE-ABS-KEY (acupuncture OR electroacupuncture OR "electro-acupuncture" OR needling OR "scalp acupuncture" OR "body acupuncture" OR "abdominal acupuncture"))

AND

(TITLE-ABS-KEY ("randomized controlled trial" OR "controlled clinical trial" OR randomized OR randomised OR placebo OR randomly OR trial OR rct))

Cochrane

#1 MeSH descriptor: [Ischemic Stroke] explode all trees

#2 stroke OR "brain ischemia" OR "ischemic stroke" OR "cerebral infarction" OR "cerebral ischemia" OR "brain infarction" OR "cerebrovascular accident"

#3 #1 OR #2

#4 MeSH descriptor: [Thrombolytic Therapy] explode all trees

#5 "tissue plasminogen activator" OR alteplase OR "rt-PA" OR tenecteplase OR reteplase OR urokinase OR streptokinase OR thrombolysis OR "thrombolytic therapy"

#6 #4 OR #5

#7 MeSH descriptor: [Acupuncture] explode all trees

#8 acupuncture OR electroacupuncture OR "electro-acupuncture" OR needling OR "scalp acupuncture" OR "body acupuncture" OR "abdominal acupuncture"

#9 #7 OR #8

#10 #3 AND #6 AND #9

Web of science

ischemic stroke (All Fields) OR stroke (All Fields) OR brain ischemia (All Fields) OR cerebral infarction (All Fields) OR cerebral ischemia (All Fields) OR brain infarction (All Fields) OR cerebrovascular accident (All Fields)

tissue plasminogen activator (All Fields) OR thrombolytic therapy (All Fields) OR alteplase (All Fields) OR rt-PA (All Fields) OR tenecteplase (All Fields) OR reteplase (All Fields) OR urokinase (All Fields) OR streptokinase (All Fields) OR thrombolysis (All Fields)

acupuncture (All Fields) OR acupuncture therapy (All Fields) OR electroacupuncture (All Fields) OR electro-acupuncture (All Fields) OR needling (All Fields) OR scalp acupuncture (All Fields) OR body acupuncture (All Fields) OR abdominal acupuncture (All Fields)

randomized controlled trial (All Fields) OR RCT (All Fields) OR randomized (All Fields) OR randomised (All Fields) OR placebo (All Fields) OR randomly (All Fields) OR trial (All Fields)

#1 AND #2 AND #3 AND #4

Embase

('ischemic stroke':ti,ab,kw OR stroke:ti,ab,kw OR 'brain ischemia':ti,ab,kw OR 'cerebral infarction':ti,ab,kw OR 'cerebral ischemia':ti,ab,kw OR 'brain infarction':ti,ab,kw OR 'cerebrovascular accident':ti,ab,kw)

AND

('tissue plasminogen activator':ti,ab,kw OR 'thrombolytic therapy':ti,ab,kw OR alteplase:ti,ab,kw OR 'rt pa':ti,ab,kw OR tenecteplase:ti,ab,kw OR reteplase:ti,ab,kw OR urokinase:ti,ab,kw OR streptokinase:ti,ab,kw OR thrombolysis:ti,ab,kw)

AND

(acupuncture:ti,ab,kw OR 'acupuncture therapy':ti,ab,kw OR electroacupuncture:ti,ab,kw OR 'electro acupuncture':ti,ab,kw OR needling:ti,ab,kw OR 'scalp acupuncture':ti,ab,kw OR 'body acupuncture':ti,ab,kw OR 'abdominal acupuncture':ti,ab,kw)

AND

('randomized controlled trial':ti,ab,kw OR rct:ti,ab,kw OR randomized:ti,ab,kw OR randomised:ti,ab,kw OR placebo:ti,ab,kw OR randomly:ti,ab,kw OR trial:ti,ab,kw)

China Database

All: (Cerebrovascular accident OR Stroke OR Cerebral infarction OR Cerebral infarction)

All: (rt-PA or alteplase or tenecteplase or reteplase or recombinant tissue plasminogen activator or urokinase or streptokinase)

All: (acupuncture OR needle therapy OR electroacupuncture OR scalp acupuncture OR body acupuncture OR acupoint OR needle OR scalp needle OR abdominal needle)

All: (randomized)
