# Supplementary material for: Early Acupuncture Following Thrombolysis in Acute Ischemic Stroke: A Meta‐Analysis of Randomized Controlled Trials on Efficacy and Safety
Source: Brain Behav. 2026 Nov 9;16(11):e71840. doi: 10.1002/brb3.71840 (PMC13746989; doi:10.1002/brb3.71840)
Supplement: Supplementary file 3 [file BRB3-16-e71840-s003.docx]

> # ==========================================

> # 手动试验序贯分析 (Trial Sequential Analysis)

> # 基于 O'Brien-Fleming alpha-spending 方法

> # ==========================================

>

> # 加载 meta 包

> library(meta)

>

> # 输入数据

> study <- c("Liu 2015","Wei 2023","Song 2022","Cao 2020","Zhang 2020",

+	"Zhang 2018","Lu 2023","Chen 2020","Yang 2023","Song 2023")

> MD <- c(-3.50, -4.40, -2.00, -2.59, -2.66,

+	-2.51, -2.47, -2.15, -5.57, -1.72)

> SE <- c(0.690, 0.571, 0.248, 0.294, 0.281,

+	0.308, 0.273, 0.232, 0.418, 0.197)

>

> # 运行 meta 分析

> meta_nihss <- metagen(TE = MD, seTE = SE, studlab = study,

+	sm = "MD", method.tau = "REML",

+	fixed = FALSE, random = TRUE)

Warning message:

Use argument 'common' instead of 'fixed' (deprecated).

>

> # ==========================================

> # TSA 参数设置

> # ==========================================

> alpha <- 0.05	# 双侧

> beta <- 0.20	# 检验效能 80%

> delta <- -2.5	# 最小临床重要差异

> k <- length(MD)	# 研究数量

>

> # 计算累积信息量 (1/方差)

> info <- 1 / (SE^2)

> cum_info <- cumsum(info)

>

> # 计算累积 Z 值 (使用随机效应模型的结果)

> # 每项研究的 Z = MD / SE

> Z_i <- MD / SE

>

> # 累积 Z 值（使用 Lan-DeMets 方法近似）

> Z_cum <- numeric(k)

> for (i in 1:k) {

+	w <- 1 / (SE[1:i]^2)

+	pooled_MD <- sum(MD[1:i] * w) / sum(w)

+	pooled_SE <- sqrt(1 / sum(w))

+	Z_cum[i] <- pooled_MD / pooled_SE

+ }

>

> # ==========================================

> # 计算 O'Brien-Fleming TSA 界值

> # ==========================================

> # 所需信息量

> var_low_het <- mean(SE^2) # 平均方差（近似低异质性下的方差）

> RIS <- ((qnorm(1 - alpha/2) + qnorm(1 - beta)) / delta)^2 * var_low_het

>

> # O'Brien-Fleming 界值函数

> t_values <- cum_info / RIS # 信息比例

> t_values[t_values > 1] <- 1

>

> # 双侧 O'Brien-Fleming 界值

> Z_OBF <- qnorm(1 - alpha/2) / sqrt(t_values)

>

>

> pdf("TSA_NIHSS.pdf", width = 8, height = 6)

> # ==========================================

> # 绘图

> # ==========================================

> plot(cum_info, Z_cum, type = "b", pch = 16, col = "blue",

+	xlab = "累积信息量", ylab = "累积 Z 值",

+	main = "试验序贯分析 (TSA) — NIHSS",

+	ylim = c(min(c(Z_cum, -Z_OBF)) - 1, max(c(Z_cum, Z_OBF)) + 1),

+	xlim = c(0, max(cum_info) * 1.2))

There were 15 warnings (use warnings() to see them)

>

> # 添加传统界值

> abline(h = qnorm(1 - alpha/2), lty = 2, col = "red")

> abline(h = -qnorm(1 - alpha/2), lty = 2, col = "red")

>

> # 添加 TSA 界值

> lines(cum_info, Z_OBF, type = "l", col = "red", lwd = 2)

> lines(cum_info, -Z_OBF, type = "l", col = "red", lwd = 2)

>

> # 添加所需信息量线

> abline(v = RIS, lty = 3, col = "darkgreen")

> text(RIS * 1.05, max(Z_cum) * 0.5, paste0("RIS = ", round(RIS, 0)),

+	col = "darkgreen", pos = 4)

>

> # 添加图例

> legend("bottomright",

+	legend = c("累积Z值", "TSA界值 (O'Brien-Fleming)", "传统界值 (1.96)"),

+	col = c("blue", "red", "red"),

+	lty = c(1, 1, 2),

+	pch = c(16, NA, NA))

Warning messages:

1: In text.default(x, y, ...) :

conversion failure on '累积Z值' in 'mbcsToSbcs': for 累 (U+7D2F)

2: In text.default(x, y, ...) :

conversion failure on '累积Z值' in 'mbcsToSbcs': for 积 (U+79EF)

3: In text.default(x, y, ...) :

conversion failure on '累积Z值' in 'mbcsToSbcs': for 值 (U+503C)

4: In text.default(x, y, ...) :

conversion failure on 'TSA界值 (O'Brien-Fleming)' in 'mbcsToSbcs': for 界 (U+754C)

5: In text.default(x, y, ...) :

conversion failure on 'TSA界值 (O'Brien-Fleming)' in 'mbcsToSbcs': for 值 (U+503C)

6: In text.default(x, y, ...) :

conversion failure on '传统界值 (1.96)' in 'mbcsToSbcs': for 传 (U+4F20)

7: In text.default(x, y, ...) :

conversion failure on '传统界值 (1.96)' in 'mbcsToSbcs': for 统 (U+7EDF)

8: In text.default(x, y, ...) :

conversion failure on '传统界值 (1.96)' in 'mbcsToSbcs': for 界 (U+754C)

9: In text.default(x, y, ...) :

conversion failure on '传统界值 (1.96)' in 'mbcsToSbcs': for 值 (U+503C)

>

> dev.off() # 新增：关闭PDF设备，保存文件

RStudioGD

2

> # ==========================================

> # 输出关键结果

> # ==========================================

> cat("\n========== TSA 结果 ==========\n")

========== TSA 结果 ==========

> cat("所需信息量 (RIS):", round(RIS, 2), "\n")

所需信息量 (RIS): 0.18

> cat("实际累积信息量:", round(max(cum_info), 2), "\n")

实际累积信息量: 119.69

> cat("信息量比例:", round(max(cum_info) / RIS * 100, 1), "%\n")

信息量比例: 65021.1 %

> cat("最终累积 Z 值:", round(Z_cum[k], 2), "\n")

最终累积 Z 值: -26.76

> cat("TSA 界值 (最终):", round(Z_OBF[k], 2), "\n")

TSA 界值 (最终): 1.96

> cat("传统界值 (双侧):", round(qnorm(1 - alpha/2), 2), "\n")

传统界值 (双侧): 1.96

>

> if (abs(Z_cum[k]) > Z_OBF[k]) {

+	cat("\n结论: 累积Z值已跨过TSA界值，样本量充分，结论可靠。\n")

+ } else if (abs(Z_cum[k]) > qnorm(1 - alpha/2)) {

+	cat("\n结论: Z值超过传统界值但未跨过TSA界值，需要更多研究。\n")

+ } else {

+	cat("\n结论: 未达到统计显著性。\n")

+ }

结论: 累积Z值已跨过TSA界值，样本量充分，结论可靠。
