# Supplementary material for: Early Acupuncture Following Thrombolysis in Acute Ischemic Stroke: A Meta‐Analysis of Randomized Controlled Trials on Efficacy and Safety
Source: Brain Behav. 2026 Nov 9;16(11):e71840. doi: 10.1002/brb3.71840 (PMC13746989; doi:10.1002/brb3.71840)
Supplement: Supplementary file 4 [file BRB3-16-e71840-s004.docx]

Efficacy and safety of acupuncture as an adjunct to thrombolysis for acute ischemic stroke: a systematic review and meta-analysis of randomized controlled trials

**Background and rationale**

Ischemic stroke (IS) accounts for approximately 70–80% of all strokes and remains a leading cause of long‑term disability and mortality worldwide. Intravenous thrombolysis with recombinant tissue plasminogen activator (rt‑PA) or urokinase within the therapeutic time window is the guideline‑recommended reperfusion therapy for acute IS. Despite timely recanalization, many patients do not achieve complete neurological recovery and may experience reperfusion injury, hemorrhagic transformation, or persistent motor and cognitive deficits.

Acupuncture has been widely used as a complementary therapy for stroke in East Asian countries. Experimental and clinical studies suggest that acupuncture may promote cerebral blood flow, reduce oxidative stress and inflammatory responses, and enhance neuroplasticity. However, the incremental benefit and safety of adding acupuncture to standard thrombolytic treatment during the acute phase remain unclear. A rigorous systematic review and meta‑analysis of randomized controlled trials (RCTs) is therefore needed to inform clinical decision‑making.

**Research objective**

Primary objective

To evaluate the efficacy and safety of acupuncture combined with intravenous thrombolysis compared with thrombolysis alone in patients with acute ischemic stroke.

Secondary objectives

To assess the effect of combined therapy on neurological impairment, activities of daily living, and motor function.

To evaluate the impact on coagulation parameters, inflammatory markers, and cerebral hemodynamics.

To explore potential sources of heterogeneity (e.g., acupuncture type, timing of intervention, treatment duration).

**PICOS inclusion criteria**

Component	Description

**Population**	Adults (≥18 years) with a confirmed diagnosis of acute ischemic stroke (based on CT or MRI) regardless of sex, stroke location, or severity.

**Intervention**	Acupuncture combined with intravenous thrombolytic therapy.

**Comparator**	Identical thrombolytic therapy without any form of acupuncture (placebo/sham acupuncture or no acupuncture).

**Outcomes**	Primary outcomes: neurological function (NIHSS) and activities of daily living (Barthel Index / BI).

**Secondary outcomes**: motor function (FMA), quality of life (SS‑QOL), mRS, inflammatory markers (IL‑6, TNF‑α, CRP), coagulation parameters (PT, APTT, fibrinogen), cerebral hemodynamics (CVR, PI), oxidative stress markers (SOD, MDA), symptom‑based scores (TCM symptom scale), and adverse events (including symptomatic intracranial hemorrhage).

**Study design**	Randomized controlled trials (RCTs).

**Exclusion criteria** (during screening)

Duplicate publications.

Non‑randomized studies (e.g., reviews, commentaries, letters, conference abstracts without full data).

Animal or in vitro studies.

Intervention group does not receive acupuncture combined with thrombolysis.

Control group receives any form of acupuncture.

Full text unavailable after reasonable attempts to contact authors.

Studies with insufficient data for effect size calculation and no response from corresponding authors.

**Search strategy**

Electronic databases (from inception to present): Pubmed, Embase, Cochrane Central Register of Controlled Trials (CENTRAL), Web of Science, Scopus, Chinese Biomedical Database (CBM), China National Knowledge Infrastructure (CNKI), Wanfang Data Knowledge Service Platform, VIP Chinese Science and Technology Periodical Database (VIP).

Search terms (combining MeSH and free‑text terms):

For stroke:

("stroke" OR "brain ischemia" OR "ischemic stroke" OR "cerebral infarction" OR "cerebral ischemia" OR "brain infarction" OR "cerebrovascular accident")

For thrombolysis:

("tissue plasminogen activator" OR alteplase OR "rt-PA" OR tenecteplase OR reteplase OR urokinase OR streptokinase OR thrombolysis OR "thrombolytic therapy")

For acupuncture:

("acupuncture" OR "electroacupuncture" OR "electro-acupuncture" OR needling OR "scalp acupuncture" OR "body acupuncture" OR "abdominal acupuncture")

For RCTs:

("randomized controlled trial" OR "RCT" OR "randomized" OR "randomised" OR "placebo" OR "randomly" OR "trial")

The search will be adapted for each database using appropriate syntax and filters. No language or publication date restrictions will be applied.

**Study selection process**

Two independent reviewers will screen titles and abstracts against the eligibility criteria. Full texts of potentially relevant articles will be retrieved and assessed independently by the same reviewers. Disagreements will be resolved by discussion or by consulting a third reviewer. A PRISMA flow diagram will be provided to document the selection process.

**Data extraction**

A standardized, pilot‑tested data extraction form will be used. Two reviewers will independently extract the following information:

Study characteristics: first author, year, age, sex, disease course, treatment measures, countermeasures, Outcome, adverse reactions.

Intervention details: type of acupuncture, needle retention time, frequency, total treatment duration, thrombolytic agent and regimen.

Missing data will be sought by contacting corresponding authors. If not obtainable, the study will be included only for outcomes with available data.

**Risk of bias assessment**

The Cochrane RoB 2.0 tool will be used to assess the risk of bias in the included RCTs. Two reviewers will independently evaluate the following domains:

Randomization process

Deviations from intended interventions

Missing outcome data

Measurement of the outcome

Selection of the reported result

Each domain will be judged as “low risk”, “some concerns”, or “high risk”. An overall risk of bias will be assigned for each study. Disagreements will be resolved by consensus or a third reviewer.

**Data synthesis and statistical analysis**

**Effect measures**

Continuous outcomes (NIHSS, BI, FMA, laboratory parameters): mean difference (MD) with 95% confidence interval (CI) will be used if the same scale is used; if different scales measure the same construct (e.g., BI), standardized mean difference (SMD) will be considered.Dichotomous outcomes (adverse events, sICH, clinical response rate): risk ratio (RR) with 95% CI.

**Meta‑analytic model**

A random‑effects model (DerSimonian‑Laird) will be used to account for anticipated clinical and methodological heterogeneity. The restricted maximum likelihood (REML) method may be used for heterogeneity variance estimation.

**Heterogeneity assessment**

Heterogeneity will be quantified using the I² statistic and the Cochran’s Q test. Thresholds: I² < 25% (low), 25–50% (moderate), > 50% (substantial). If I² > 75% and the direction of effect is inconsistent, a narrative synthesis will be considered instead of meta‑analysis.

**Subgroup analyses (if sufficient data available)**

Type of thrombolytic agent (rt‑PA vs. urokinase)

Timing of acupuncture initiation (≤24h after thrombolysis vs. >24h)

Total treatment duration (≤2 weeks vs. >2 weeks)

**Sensitivity analyses**

Leave‑one‑out analysis to assess influence of individual studies.

Exclusion of studies with high risk of bias.

Fixed‑effect model as an alternative to test robustness.

**Publication bias**

Funnel plots will be examined for asymmetry if ≥10 studies are included. Egger’s linear regression test (P < 0.10 indicating significant asymmetry) will be performed for the primary outcome.

**Software**

All analyses will be conducted using R software (version ≥4.2) with the meta and metafor packages.

**Presentation of results**

Results will be presented in accordance with the PRISMA 2020 statement. A forest plot will be generated for each meta‑analysis. A summary of findings table will be created using GRADE methodology to rate the certainty of evidence for primary and key secondary outcomes.
